# Risk of Infective Endocarditis Post-transcatheter Pulmonary Valve Replacement Versus Surgical Pulmonary Valve Replacement: A Systematic Review

**DOI:** 10.7759/cureus.48022

**Published:** 2023-10-31

**Authors:** Ethan Slouha, Lashawnd L Johnson, Arani Thirunavukarasu, Hanin Al-Geizi, Lucy A Clunes, Theofanis F Kollias

**Affiliations:** 1 Anatomical Sciences, St. George's University School of Medicine, St. George's, GRD; 2 Pharmacology, St. George's University School of Medicine, St. George's, GRD; 3 Pharmacology, St George's University School of Medicine, St George's, GRD; 4 Microbiology, Immunology and Pharmacology, St. George's University School of Medicine, St. George's, GRD

**Keywords:** percutaneous pulmonary valve replacement, percutaneous pulmonary valve implantation, surgical valve replacement, pulmonary valve replacement, infective endocarditis

## Abstract

Pulmonary valve replacement (PVR) is the most common cardiac operation in adult patients with congenital heart disease (ACHD). It can improve right ventricular outflow tract (RVOT) obstruction, typically due to pulmonary valve stenosis or regurgitation. PVR can be performed surgically (open-heart) and through a transcatheter (percutaneous) method, which is minimally invasive and is associated with shorter hospitalization stays. However, following PVR, infectious endocarditis (IE) can complicate the recovery process and increase mortality in the long term. IE is a rare but deadly multi-organ system condition caused by microorganisms traversing the bloodstream from a specific entry point. It can have many presentations, such as splinter hemorrhages, fevers, and vegetation on valves that lead to stroke consequences. This paper aims to evaluate the differences in the rate, etiology, manifestations, treatment, and outcomes of IE following surgical and transcatheter PVR, as the goal is to perform a procedure with few complications. In both approaches, *Staphylococcus aureus* was the most common microorganism that affected the valves, followed by *Streptococcus viridians*. Research has shown that surgical pulmonary valve replacement (SPVR) has a decreased risk of IE following surgery compared to TPVR. However, TPVR is preferred due to the reduced overall risk and complications of the procedure. Despite this, the consensus on mortality rates does differ. Future research should consider the type of valves used for transcatheter pulmonary valve replacement (TPVR), such as Melody valves versus Edward Sapien valves, as their IE rates vary significantly.

## Introduction and background

Pulmonary valve replacement

Pulmonary valve replacement (PVR) is primarily used to improve the right ventricular outflow tract (RVOT), which is most commonly seen in patients following the repair of the tetralogy of Fallot [1]. RVOT dysfunction usually arises from pulmonary stenosis and sometimes pulmonary regurgitation, eventually leading to right ventricular (RV) failure and arrhythmias. There is no defined time when PVR should be performed, so physicians rely on the functioning of the RV, such as moderate pulmonary regurgitation or an RV systolic pressure> 2/3rd of the systemic pressure [1]. Balzer defines the PVR as being performed if the following criteria are met: RV end-systolic volume index (RVESVi) > 82 mL/m^2^, RV end-diastolic volume index (RVEDVi) >150 mL/m^2^, progressive tricuspid regurgitation with a dilated RV, significant RV dysfunction such as RV ejection fracture <45%, and sustained arrhythmias combined with a dilated RV [1]. Patients with these criteria may still be asymptomatic, so continuous observation must be done when a problem is identified. There are two ways to perform PVR: surgical and transcatheter (percutaneous).

Surgical pulmonary valve replacement

Surgical pulmonary valve replacement (SPVR) is performed through open-heart surgery using a sternotomy to access the damaged valve. The surgeon removes the pulmonary valve and replaces the valve with either a mechanical, bovine, porcine, or human cadaver valve [2]. Factors such as medication and the longevity of the valve must be important considerations prior to valve replacement. Factors such as medications must be considered following the procedure, as well as the longevity of the valve. Mechanical pulmonary valves require lifelong anticoagulants to prevent valve thrombosis, while biological valves undergo wear and tear to the point of breaking down [2]. The trend over the past decade has trended towards moving from SPVR to transcatheter (percutaneous) pulmonary valve replacement (TPVR) in the majority of patients. This decrease in SPVR is due to the procedure carrying more detrimental risks such as increased mortality overall, the risk of recurrent pulmonary regurgitation, and the risk of readmission [3].

Transcatheter (percutaneous) pulmonary valve replacement

Surgeons are starting to prefer using TPVR as a method of PVR, as studies have indicated fewer postoperative complications and shorter hospital stays. TPVR has also led the way in advancing approaches to the intervention [4]. The main obstacle observed with this procedure currently is the large delivery systems required, optimal timing to prevent complications of RVOT failure, and the need for a landing zone through a conduit or bioprosthetic valve [4]. Some of the most common valves used in PVR are the Melody valve (Medtronic, Inc., Dublin, Ireland), the Sapien valve (Edwards Lifesciences, Inc., Irvin, CA), and the bovine jugular vein (BJV) [4]. Each varies in their inclusion and exclusion criteria, where Melody is more about the age of the patient, while Sapien is more about the patient's weight [4]. The implantation is done by inserting a catheter into a large blood vessel, usually in the groin or chest, and guiding it to the heart. The new valve goes through the catheter, and a balloon at the tip of the catheter expands to push the new valve into place [2]. Following either SPVR or TPVR, one complication they share is an increased risk of infective endocarditis (IE), which has detrimental consequences such as death.

Infective endocarditis

IE is a rare but life-threatening condition with long-lasting effects even when the patient is treated, and only 50% of people survive 10 years following diagnosis [5,6]. IE can occur in patients with no structural heart disease due to microorganisms infecting and traversing the bloodstream. The risk of IE increases drastically after dental procedures, IV drug abuse, and procedures like cardiac implantation due to bacteria entering the bloodstream via the skin [7]. Those with damaged or implanted heart valves are at a greater risk because it leads to the classical sign of vegetation on the valves. Vegetations are masses composed of platelets, infecting organisms, and fibrin held together by agglutinating antibodies that the infecting bacteria produce [8]. Once IE sets in the body, it can involve almost any organism in its path. The modified Duke criteria are used to diagnose IE; they consist of major clinical criteria, such as positive blood culture, vegetation, or new valvular regurgitation, and minor clinical criteria, such as predisposing conditions and vascular phenomena [5]. Vegetations can occur in damaged native and prosthetic heart valves and usually happen at the line of closure of the valve leaflets in combination with reduced blood flow, allowing bacteria to adhere to the valves [5]. Prosthetic valves are at greater risk and can occur in the initial weeks after the procedure due to skin bacteria entering the bloodstream or direct intraoperative contamination [9]. The most common microorganism involved in IE in both native and prosthetic valves is *Staphylococcus aureus*.

Intravenous antibiotics are recommended for six to eight weeks, but the type of antibiotic used depends on the underlying bacterial cause [7]. If no antibiotic is started, the mortality risk is up to 20% within the first 30 days [7]. There are some instances where antibiotics alone do not control the infection, and surgery is required to remove or fix vegetation and abscesses and even remove implanted valves or devices [7]. To an extent, when surgery is not advised despite indications, these patients may end up on long-term oral suppressive antibiotic treatment, which could continue for a median of 277 days [10]. Despite the improvement of therapy and treatment, reinfection with IE is found to be up to 5-10% [11].

Aim

This paper aims to assess and compare the incidence, risk factors, etiology/microorganism, symptoms, treatment, outcomes, and complications/mortality of IE following both SPVR and TPVR. The goal is to review whether data suggest an increased risk of IE with either SPVR or TPVR and whether this affects long-term outcomes for the patient, thus guiding which procedure is preferred.

## Review

Methods

A systematic and exhaustive literature search was performed using ScienceDirect, PubMed, and ProQuest catalogs from January 1, 2003, to December 31, 2023. The search keywords included "infective endocarditis after surgical pulmonary valve replacement," "infective endocarditis after transcatheter pulmonary valve replacement," and "infective endocarditis after pulmonary valve replacement." The electronic inquiry concentrated on peer-reviewed experimental publications that were in line with the purpose of this article. Publications published before 2003, those not written in English, and duplicates were excluded from the screening process. Once articles were acquired, four independent co-authors evaluated the information. The publications acquired in the search were evaluated based on the study type, abstracts, titles, and full-text accessibility. The initial inquiry into the three catalogs resulted in 46,067 publications. The chosen publication was further tailored based on keyword specifics and the information provided in the abstract. A total of 19 publications were found to be within the scope of this paper according to the following criteria.

Inclusion Criteria

The following inclusion criteria were used: publications published between 2003 and 2023, written in English, conducted on humans, focused on IE following either SPVR, TPVR, or both, were experimental, cohort, case-control, meta-analyses or observation, full-text, and peer-reviewed.

Exclusion Criteria

**Figure 1:**
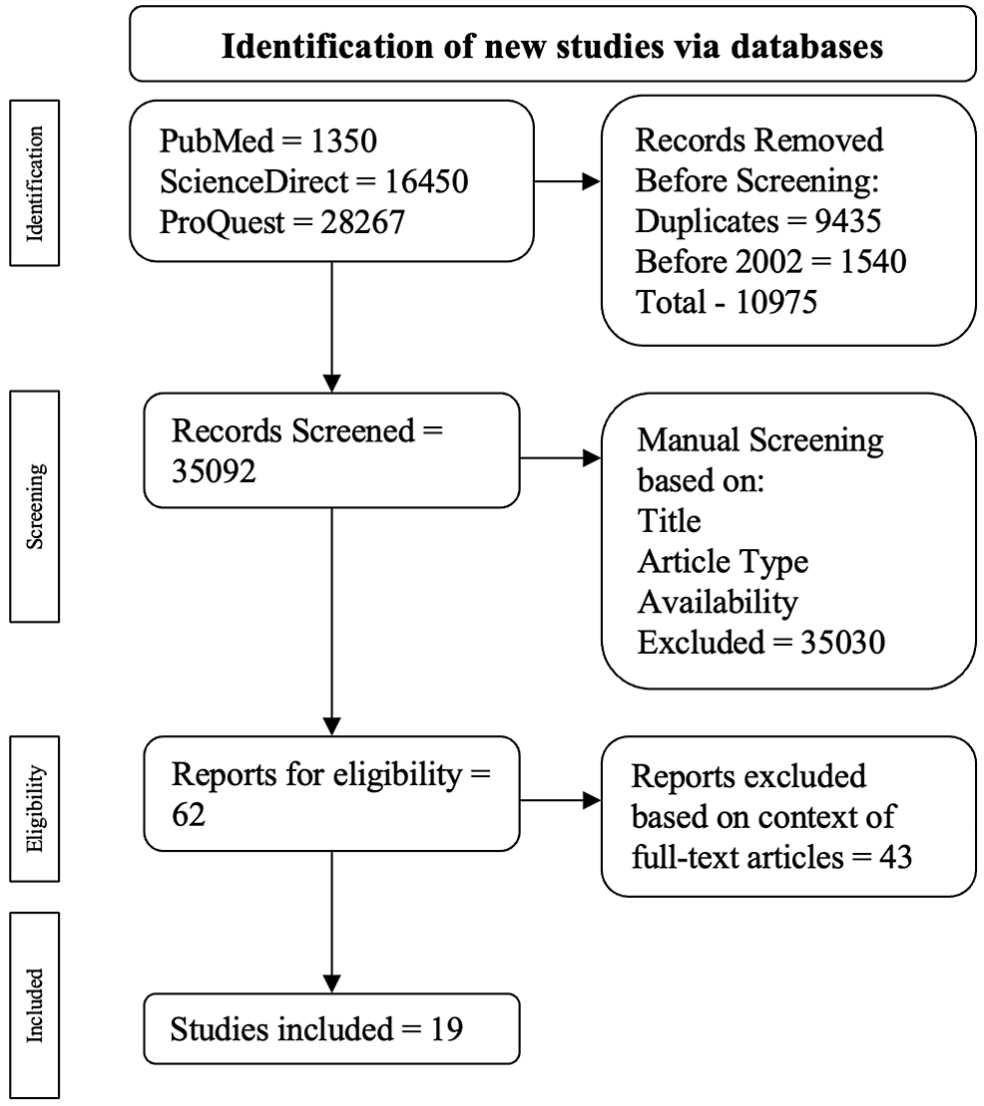
Visual representation of the algorithm used for the inclusion and exclusion criteria. The pathway used for the inclusion and exclusion criteria came from the PRISMA review [12].

Exclusion criteria included narrative reviews, systematic reviews, and case series or reports. All duplicates and non-full-text publications were excluded as well. The publication’s inclusion and exclusion development is drawn out in Figure 1.

Bias

All studies were evaluated for bias. A few articles did not disclose their entire protocol and weighed slightly on the overall bias. A moderate GRADE scale was concluded. To assess the individual risk of bias, the tool used was GRADE (grading of recommendation, assessments, development, and evaluations), which evaluates flaws like imprecision, indirectness, and publications.

Results

There were 46,067 publications found: 1350 from PubMed, 16,450 from ScienceDirect, and 28,267 from ProQuest. Among the exclusions, 9435 were duplicate publications, and 1540 were published before 2003. This resulted in 10,975 publications being excluded during the automatic screening process, leading to 35,092 publications for manual screening. Publications were then manually evaluated based on the title, availability, abstract, and study type, resulting in 62 articles being checked for eligibility via full-text analysis. Ultimately, 19 articles were used.

IE is a rare occurrence following PVR. Variations in the incidence of IE occur between SPVR and TPVR, with SPVR having a lower incidence. The most common organism that leads to IE in both surgeries is S. aureus, followed by Streptococcus viridians. There is also a similarity in how the condition is expressed, such as high fever, which can occur before a positive culture, pleuritic chest pain, and most importantly, the majority develop vegetation along the closure line of the valve leaflets. There is also speculation that the risk of IE varies depending on the type of valve used. It is observed that Melody valve replacement, using TPVR, had the highest incidence of IE compared to any other kind of valve. As mentioned before, SPVR has more associated complications following surgery than TPVR, which is why it is still preferred to SPVR, especially considering IE is still a relatively rare phenomenon. Table 1 depicts the articles gathered from the inquiry for this systematic review.

Discussion

Incidence

When considering IE following PVR in general, Stammnitz et al. observed that IE was seen in 4.8% of patients following a median follow-up of 10 years [13]. The median time to develop IE post-prosthetic valve replacement (SPVR and TPVR) ranges from 48 months to up to five years, specifically for Melody valves [14]. IE does not typically develop during the first year after implantation, but early IE does occur [15]. Of the patients in the study by Stammnitz et al., 21.4% had early IE (within one year of PVR), and 78.6% had late IE [13].

The annual incidence of IE following the SPVR procedure in the study by Groning et al. is 0.69% at one year and 0.7% at five years. Specifically with Contegra graft implantation, the annual incidence was 0.97% at one year and 1.12% at five years post-valve replacement, and the homograft placement was 0.4% at one year and 0.27% at five years [16]. Homografts were 100% survival-free from IE at one year and 99% at five years following implantation, whereas Contegra grafts were 99% survival-free from IE at one year and 94% at five years following implantation [16]. Van Dijck et al. observed that 2.4% of patients who received a homograft developed IE after 5.7 years, compared to 20.4% who received a Contegra graft after 4.8 years [15]. Beckerman et al. observed that 10% of patients who had undergone a BJV graft developed endocarditis 7.5 years following surgery [17].

Studies have indicated that the cumulative IE incidence following TPVR ranges from 5.14% to 11.8% [15,18-20]. Hasacoet et al. found that the overall incidence of IE post-PPVI was 3.9% per person-year [21]. The incidence was 9.5% at five years and 16.9% at eight years following TPVR [20]. McElhinney et al. observed that the annualized incidence rate of TPV-related IE was 0.88% per patient per year. In contrast, a later study observed that the annualized incidence rate of TPV-related IE was 2.4% per patient per year [20,22]. Malekzadeh-Milani et al. observed a comparable rate of 3.6% per patient-year, whereas Tanase et al. found that the annualized incidence was up to 10.9% [19,23]. The median time of developing IE after TPVR was an average of 2.4 years, and the median age of IE development was 17.9 years in one of the studies [18,22-25]. The average estimated freedom from the diagnosis of endocarditis was 96.65% at one year and 87.6% at five years [15,19,22]. Patients who developed IE tended to be older than those without IE, with a mean age at implantation of 29.8 ± 16.3 years [26].

It is essential to note the different types of transcatheter valves used, as many studies have indicated that Melody valves were more likely to lead to IE following TPVR, having an incidence between 2.7% and 6.6% [13,23,26,27]. 11.7% of patients who had undergone TPVR with a Melody valve had developed IE in the study by Malekzadeh-Milani et al. [26]. The incidence of endocarditis was 2.4 per 100 patient-years in patients with a Melody valve, compared to 0.8 per 100 patient-years in those with a Sapien valve; a significant difference was found in the study by Hascoet et al. [21,24]. The annual incidence of IE for patients with a Melody valve was 6.96% at one year and 2.89% at five years [16,23]. Melody valves, which accounted for 15.1% of pulmonary valve replacements, showed a higher incidence of IE of 7.5% [13]. The interval between the onset of IE and Melody valve implantation was 1.5-2.6 years [19,27].

The incidence of IE was significantly lower in patients who received SPVR than those who received TPVR [28]. 1.4% of patients who underwent SPVR developed IE, compared to 3.4% of patients who underwent TPVR [29]. Malekzadeh-Milani et al. concluded that IE was significantly higher in patients with TPVR than SPVR [28,30]. O’Donnell et al. noticed a drastic difference, with only 2% of SPVR patients developing IE compared to 16% who underwent TPVR [31]. There was 94.0% four-year freedom in the SPVR group compared to 84% in the TPVR group [29]. IE occurred much sooner in the TPVR group than in the surgical group [30]. However, Cedkirdecki et al. found that follow-up time post-TPVR and SPVR surgeries did not significantly differ in the risk of IE [28].

Risk Factor

The risk of developing IE following PVR significantly increases in a patient who receives multiple implants, which may be due to the formation of microscopic lesions that occurred during their procedure, resulting in turbulence leading to non-bacterial thrombotic endocarditis, which then turns into infective after any transient bacteremia [13,16]. Following SPVR, the risk of developing IE was non-significantly increased in patients who received a Contegra graft compared to homografts [16]. Patients with a history of IE were up to seven times more likely to develop IE following SPVR [30]. It is essential to consider that Cekirdekci et al. observed that out of 1,000 SPVR patients who develop IE, at least 30 cases of IE could be prevented. Still, independent risk factors need further evaluation [28].

Certain factors associated with a higher rate of IE following TPVR were gender, balloon post-dilation, smaller RVOT, conduits, stenotic lesions, history of IE, discontinuation of antiplatelet or anticoagulant therapy and percutaneous intervention, dental care, or non-cardiac surgery after TPVR [18,21]. Patients who developed IE following TPVR tended to be older than those without IE, with a mean age at implantation of 29.8 ± 16.3 years [26]. McElhinney et al. observed that patients with a higher preimplant RVOT gradient and those with a higher early post-implant RVOT gradient were significantly associated with a shorter time to TPVR-related IE [20]. The increased risk of IE with Melody valves could be due to the residual right ventricle-to-pulmonary artery pressure gradient during the percutaneous pulmonary valve implantation [13]. This is compared to Sapien valves, where IE could be caused during implantation, which may be why IE can show up as early as two months following the procedure [27]. The process of harvesting, descaling, and denaturing the bovine pericardium for Sapien valves could be a reason for their decreased susceptibility to bacterial colonization compared to Melody valves [27].

In comparing both procedures, the risk of developing IE was significantly increased in patients who received a Melody TPVR valve compared to homografts receiving SPVR [16]. Patients in the surgical group were noted to be younger than those receiving specifically a Melody valve, and those who underwent a Melody valve implantation were discovered to have had more previous surgeries [30]. Another risk factor associated with IE in patients who underwent TPVR was a higher gradient across the RVOT before surgery, and they tended to have a conduit [29]. Particular grafts are also performed, either surgical or transcatheter, such as the bovine jugular vein valve, and this specific valve has been shown to have the highest risk of IE, regardless of the placement method [13].

Etiology/Microorganism

Common microorganisms involved in IE following PVR were coagulase-negative Staphylococci, *Staphylococcus aureus*, and Streptococcus [13,14]. The most common organisms found in IE patients following SPVR were *Staphylococcus aureus* (including methicillin-sensitive), Enterococcus, and *Streptococci viridians* [17,30,31]. The most common pathogenic bacteria causes of IE in TPVR were *Staphylococcus aureus*, HACEK, and *Streptococcus viridians* [18-20,24,28]. Other bacteria that showed up infrequently were *Aerococcus viridians*, *Streptococcus mitis*, *Haemophilus influenzae*, *Corynebacterium striatum*, and *Coxiella burnetti* [19,21]. Immunocompromised patients were more likely to have a higher rate of culture-negative endocarditis, but this was not significant [25]. Precipitating factors of IE following TPVR that were identified in 25 cases were unprotected dental procedures/orthodontics/oral trauma, infected wounds, cat scratches/bites, paranasal sinusitis, dermatophytosis complex, nail-biting and bad hygiene, cystitis, hemodialysis, upper respiratory tract infection, signs of pneumonia, gastroenteritis, and tattooing [23]. The most common microorganism involved in IE after implantation of a Melody valve is *Staphylococcus epidermidis* [31]. IE developed after Melody valve implantation could be caused by bloodstream infections and avoided by maintaining proper personal hygiene and wound care; psychomotor retardation could make an individual more susceptible [15,26,27]. Between SPVR and TPVR, there was no variation between the significant organisms involved.

Manifestations

IE symptoms persisted for a median of 21 days before the official diagnosis, and common symptoms were fever, fatigue, and splinter hemorrhages [16,17]. The effect of IE varied between aortic regurgitation, oscillating masses, abscesses related to the aortic valve, and mitral valve regurgitation following pulmonary valve homograft implantation [16]. Vegetations are expected in at least half of SPVR patients who developed IE; most were consistent with Duke’s criteria [17,29-31]. Groning observed that in those who underwent homograft placement, 80% were diagnosed with IE with vegetation present, compared to 50% in Contegra graft patients [16].

Not all patients met the Modified Duke Criteria. Still, physicians had defined them as having IE following TPVR so long as some criteria were met and the patient was symptomatic and deteriorating [18,19,21]. In the study by McElhinney et al., of the 16 patients diagnosed with definite IE, 6 met the criteria of TPV-related IE: 3 had vegetation on the TPV, 2 had new or progressive hemodynamic dysfunction of the TPV, and 1 had both [20]. The primary presentation of IE was a fever seen in 77.3% of patients in the study by Bos et al. and pleuritic chest pain seen in patients in the study by O’Donnell et al. [18,31]. McElhinney et al. observed that among those patients whose first endocarditis episode was classified as TPV-related, 24% had no objective evidence of the involvement of TPV [22]. Vegetations were found in 40.9% of patients via transesophageal echocardiography, and most of those were observed in the right RVOT [18,21,30].

Treatment

While focusing on PVR in general, Isaza et al. found in their cohort that 75% of patients underwent surgical management while 25% underwent medical management alone. Treatments for IE in surgical patients vary based on major and minor symptoms, ranging from the removal of infected conduits to medical treatment with antibiotics and even a heart transplant in cases of intractable endocarditis [30]. Treatment of IE was split between medical treatment and surgery, as with most IE patients. Medical treatment consisted of antibiotics and antiplatelet therapy [19,22,30]. When antiplatelet therapy, such as aspirin, was discontinued, there was a statistically significant association with severe IE development [19]. Surgical treatment ranged from premature interventional cardiac catheterization due to severe right ventricular outflow tract obstruction to complete surgical valve replacement [18,19,21,30]. In both SPVR and TPVR, the treatment greatly depends on the presented symptoms, but when similar symptoms are present, the patients usually receive the same treatment [18,19,21,30].

Outcomes

Isaza et al. observed that 41.7% of patients died from IE following PVR, and of those who died, 30% were being treated in the hospital [14]. The probability of survival in patients developing IE after SPVR at 12 months was 99.5%; at 24 months, it was 93.8%; and at 36 months, it was 93.8% [30]. The probability of survival from IE following TPVR is estimated to be 98.9% at 12 months, 96.8% at 24 months, and 92.3% at 36 months [30]. Malekzadeh-Milani et al. observed that the cumulative probability of survival without any cardiovascular events for IE patients following TPVR was 20% at 20 months [26]. Mortality rates varied between studies, but most causes were due to septic shock [18,19,21,30]. In an early study, McElhinney et al. found a mortality rate of 4% in patients who developed IE following TPVR. In contrast, a later study of a larger population noted that the cumulative incidence rate of death from IE following PVR was 0.8% at five years and 0.9% at eight years [22,24]. However, Malekzadeh-Milani et al. noted a mortality rate of 14% [19]. McElhinney et al. observed that younger patients with endocarditis were no more likely to die than older patients [22]. On competing risk analysis, inclusive of reoccurrence, explant due to endocarditis, and death associated with endocarditis as competing outcomes, the estimated cumulative incidence of recurrent IE was 7.5% at three years after the first endocarditis diagnosis in TPVR patients and 11.7% at five years [24].

Malekzadeh-Milani et al. observed that overall mortality at the final follow-up was 19%, with 37% of those who underwent TPVR compared to 13% who underwent SPVR [30]. However, most recently, Cekirdekci et al. observed that the overall mortality rate was similar between SPVR and TPVR, possibly due to many factors, such as improved implantation methods [28]. Some suggest that the type of valve may be the underlying cause of varying rates of IE, as the incidence varies in the different grafts used in TPVR procedures [28]. Nonetheless, there is still a variable difference between SPVR and TPVR overall because of what surgeons can use for the replacement. Implementing better lifestyle habits before the implantation or replacement could reduce the incidence of IE [15].

One limitation of the study was the number of articles covering IE that followed SPVR, as an exhaustive search only found a few compared to TPVR articles. Another limitation is that the majority of papers focus specifically on the Melody valve and not just the TPVR procedure. There was enough information to highlight important information and trends, and most documents agreed on such trends and information. While the occurrence of IE is relatively rare, it is still a life-threatening condition that warrants further studies to prevent and reduce it. Further studies should be performed on IE following SPVR because there may be a reduced risk of IE following the procedure. Another topic to gain insight into would be observing the different transcatheter valves and determining which is superior concerning decreased IE rates.

**Table 1:** Summary of findings from all articles gathered for this systematic review per PRISMA [12]

	Author	Country	Design and study population	Findings	Conclusion
1	Beckerman et al. [17]	USA	Retrospective study (n = 228)	10% of bovine jugular vein conduits developed infective endocarditis around 7.5 years after surgery. Symptoms lasted a median of 21 days before the diagnosis of IE. *Streptococci viridans* was the most common infectious agent at 52%. Freedom from IE was 97% at 5 years and 77% at 10 years. Bovine jugular vein conduits had a significantly higher incidence of IE than homografts.	Late IE is significantly high in BJV patients, which requires more monitoring and evaluation, especially if the graft has been in for more than 7 years.
2	Bos et al. [18]	Belgium	Retrospective study (n = 240)	There were 23 episodes of IE in 22 patients out of 240, with a male predominance (86%). The median age at IE episode was 17.9 years, and the median time following PVR to IE. It was 2.4 years. Streptococcal species were the most common cause of IE in these patients at 43%. Only 10 patients presented with vegetations seen from echocardiography. There were no IE-related deaths.	9.2% of patients who underwent percutaneous pulmonary valve implantation developed IE with a male predominance, and no case ended in death.
3	Cekirdekci et al. [28]	Turkey	Meta-analysis (n = 4706)	Patients who underwent TPVR had a significantly higher risk of developing IE than those who underwent SPVR, out of 1,000 SPVR patients, 30 cases of IE would be prevented.	TPVR has a greater risk of IE development compared to SPVR
4	Hascoet et al. [21]	France	Retrospective study (n = 79)	PPVI was performed in 79 patients (Melody valve, 40.5%; Sapien valve, 59.5%), 8 (10.1%) were diagnosed with IE at a median of 1.8 years after surgery. All 8 of the cases occurred after Melody PPVI. The incidence of IE post-Melody PPVI was 5.7% per person-year. The Kaplan-Meier cumulative incidence of IE with Melody PPVI was 24.0% after 4 years and 30.1% after 6 years, compared with 0.0% with the Sapien PPVI after 4 years.	IE post-PPVI may be less familiar with the Sapien Valve than the Melody valve.
5	O’Donnell et al. [31]	New Zealand	Retrospective Cohort (n = 25)	Of the 25 patients that underwent Melody implantation, two presented with life-threatening endocarditis and obstructive vegetation at 14- and 16-month post-implant. Two other patients presented with subacute endocarditis approximately 5.5 years post-implant. Of the 178 surgical pulmonic bioprostheses, four patients developed endocarditis out of the 25 in the Melody group.	The Melody valve is a good alternative; however, the Melody group had a higher risk for endocarditis than those in a contemporary surgical pulmonary implant cohort.
6	Rinaldi et al. [25]	USA	Retrospective Cohort (n = 328)	The incidence of IE in patients at this specific institution was 8% over a median follow-up of approximately 2.92 years. Patients with any immunocompromised condition were shown to be more likely to develop IE.	The high rate of endocarditis in transcatheter pulmonary valve replacement patients, especially those with Melody valves, is concerning.
7	Stammnitz et al. [13]	Germany	Retrospective Cohort (n = 1170)	IE occurred in 4.8% of the patients during the follow-up. Of the 241 patients with a Melody valve implant, 18 developed IE. There was a notable increase in the IE risk with those with a bovine jugular valve, Contegra valve, and Melody valve.	Bovine jugular vein valves seem to be associated with the highest risk of IE regardless of the deployment mode (surgical or percutaneous).
8	Isaza et al. [14]	USA	Retrospective cohort study (n = 2,124)	There were 2,124 cases of IE during the study period, with 24 (1.1%) of patients having had PoV IE. Many issues of PoV IE occurred in patients with prosthetic valves (54.2%). Coagulase-negative Staphylococci species were found to be the most common micro-organisms. 75% of the patients required surgical intervention. The median follow-up was noted to be 2.8 years. Overall, there was a significant statistical difference in the survival among the groups (p=0.03), mainly because of better outcomes for patients with CHD than those with miscellaneous risk factors.	As a result of the 16-year series, a large proportion of patients with PoV IE required surgical intervention. Patients with both PoV IE and CHD had a better survival rate than those with miscellaneous risk factors at a median follow-up of 2.8 years.
9	Groning et al. [16]	Denmark	Retrospective cohort study (n = 311)	The annual incidence of IE at 1 year in homografts was 0.4%, in Contegra grafts was 0.97%, and in Melody valves was 6.96%. Five years following the procedure, the incidence of IE in homografts was 0.27%, in Contegra grafts 1.12%, and in Melody valves 2.89%. Compared to homografts, the hazard rates for Contegra grafts and Melody valves were 3.2 and 11.89, respectively.	Bovine pulmonary conduits, such as Melody valves, were more likely to lead to IE following PVR than homografts and Contegra grafts.
10	Malekzadeh-Milani et al. [30]	USA	Retrospective cohort study (n = 31)	During the study period, 31 patients were noted to have right-sided endocarditis; 13 had had valves implanted during the study, and 18 before. The implantation-endocarditis time interval was shorter in the patients in the PPVI group. Those with a history of IE were found to correlate with endocarditis. IE was more frequent in those patients with bovine jugular vein valves compared with others. The probability of survival at 12, 24, and 36 months was 99.5%, 93.8%, and 93.8% in the surgical group compared to 98.9%, 96.8%, and 92.3% in the PPVI group, respectively (P=0.6).	There was noted to be a higher incidence of endocarditis in patients with PPVI compared to those with surgical pulmonary valves. However, despite a higher incidence of endocarditis in the PPVI group, the probabilities of survival and event-free survival were like those in the surgical group.
11	Tanase et al. [23]	Germany	Retrospective cohort study (n = 226)	The annual incidence of IE for all patients who received a valved stent in their right ventricular outflow tract was 1.9%. Freedom from IE 8 years after their percutaneous pulmonary valve implantation was approximately 87%. Finally, the probability of valve removal due to IE was about 7% after 8 years.	The incidence of IE after percutaneous pulmonary valve implantation is acceptable and similar to surgically implanted biological valves. Despite IE in some instances, freedom from reoperation was high, and the valves performed well during long-term follow-up.
12	Malekzadeh-Milani et al. [19]	France	Observational Study (n = 43)	The cumulative IE incidence was 11.8%. The annualized IE incidence was 3.6 %. Freedom from IE was 96.3% and 85.8% at 12 months and 60 months, respectively. The mean period between PPVI and IE was 2.6 years. When comparing survival rates between the IE group and non-IE group, death along with cardiovascular events were statistically higher in the IE group.	Melody valve IE is noted to be a severe complication post-PVVI. The annualized IE incidence in this study was similar to those reported in other studies. The outcome has been improved with fast diagnoses and adequate treatment, and an unfavorable outcome is mainly associated with *S. aureus*.
13	McElhinney et al. [20]	USA	Retrospective cohort study (n= 311)	The 3 trials included 311 patients followed for 687.1 patient-years. The annualized rate of a first episode of IE was 2.4% per patient-year, and TPV-related IE was 0.88% per patient-year. All the patients were treated with intravenous antibiotics.	Bacterial endocarditis has occurred in all the prospective multicenter studies of the Melody valve in North America and Europe. However, most cases did not involve TPV and responded to antibiotics. More data is necessary to understand the risk factors in this population completely.
14	Van Dijck et al. [15]	USA	Retrospective study (n = 677)	Melody valve stents were placed in 107 patients, of whom 7.5% developed IE during a follow-up of 2 years. Homografts were implanted in 517 patients, where 14 developed IE during their median follow-up of 6.5 years. Contegra grafts were placed in 53 patients, where 11 developed IE during their follow-up of 8.8 years.	The Contegra and Melody implantations have been shown to have a higher incidence of IE than homografts. This indicates that IE could be a significant threat to long-term conduit function.
15	McElhinney et al. [22]	USA	Retrospective cohort study (n = 309)	The annualized incidence rate of endocarditis was 3.1% per patient-year, and TPV-related endocarditis was 2.4% per patient-year. By multivariable analysis, age ≤12 years at implant and the immediate post-implant peak gradient ≥15 mmHg were noted to be associated with the development of endocarditis and with the development of TPV-related endocarditis.	Endocarditis is a vital adverse outcome post-TPVR in children and adults with post-operative congenital heart disease involving RVOT. The continuous efforts to understand, prevent, and optimize the management of this complication are essential to having the best outcome of TPV therapy.
16	Lehner et al. [27]	USA	Meta-Analysis (n = 4117)	The sensitivity analysis shows the incidence rate was 252.1 for Melody valves and 2.7 for Sapien valves.	The study concludes that there is an essential difference in the risk of IE following percutaneous pulmonary valve implantation. To decrease the risk of IE after percutaneous pulmonary valve implantation, it seems beneficial to select Sapien valves instead.
17	Malekzadeh-Milani et al. [26]	France	Prospective Cohort (n = 86)	The estimated freedom from IE was 91% at 50 months. Most patients with IE underwent additional unprotected invasive procedures and discontinued antiplatelet therapy. The overall probability of survival without cardiovascular events for IE patients was approximately 20% at 20 months compared to non-IE patients, who had a 98.1% survival. Death was also statistically associated with IE.	Early and late-onset Melody valve IE has become a catastrophic percutaneous pulmonary valve implantation complication. Abrupt discontinuation of aspirin and additional unprotected invasive procedures noted during follow-up seem to be significant indicators of IE. Due to its rapidly progressive nature, there should be no delay in aggressive invasive management.
18	McElhinney et al. [24]	USA	Retrospective Cohort (n = 2,476)	A cumulative incidence of 9.5% at 8 years and an annualized incidence of 2.2 per 100 patient-years. *Staphylococcus aureus* and *Streptococcus viridans* species accounted for 56% of the cases. Endocarditis was severe in 44% of the patients, and 12 (6.6%) died, all nearly infected with Staphylococcus aureus.	The incidence of endocarditis in this multicenter study was consistent over time and with prior small studies. The findings from this study and ongoing efforts to understand and alleviate risk will be vital to improving the lifetime management of patients with heart disease involving the RVOT.
19	Lluri et al. [29]	USA	Retrospective study (n = 342)	Patients who underwent TPVR were likely to have a history of IE compared to SPVR and RV to PA conduit. 1.4% of patients who underwent SPVR developed IE compared to 3.4% of patients who underwent TPVR. There was a 94.0% 4-year freedom in the SPVR group compared to 84% in the TPVR group. A risk factor associated with IE in TPVR patients was a higher gradient across the RVOT before surgery, and they tended to have a conduit.	TPVR patients were not at an increased risk of developing IE compared to patients who underwent SPVR but were more likely to have a prior history of IE. The highest risk was associated with stenotic RV to PA conduits.

## Conclusions

IE is a rare but life-threatening complication that can occur following SPVR or TPVR. PVR is performed to help restore healthy blood circulation in the heart, alleviate symptoms, and extend the lifespan. This procedure is typically performed on young individuals, typically under 30, primarily to correct congenital cardiac anomalies. IE occurs in individuals with normal cardiac function, but PVR, both SPVR and TPVR, increases the risk due to exogenous implantation. Most research has found that TPVR has an increased risk of IE following the procedure compared to SPVR. More specifically, the Melody valve implanted through TPVR has the greatest risk. The predominant causative microorganism in IE is S. aureus for TPVR and SPVR. Symptoms include high fever, chest pain, and vegetation along the closure line of the valve leaflets, but these do not necessarily occur in all patients. Despite the decreased incidence of IE following SPVR compared to TPVR, TPVR is still preferred due to the reduced prevalence of more common complications, both severe and not severe. Transcatheter procedures have been shown to succeed in their intended purpose overall, but that does not necessarily mean they are always safer. While IE’s incidence following PVR is considerably small, the extensiveness of IE still warrants further research in trying to reduce its presence. Research needs to evaluate the long-term effects of PVR-associated IE in young patients who undergo the procedure, compare TPVR valves, and potentially come up with new ways to perform the procedure to prevent long-term complications.
